# Cardiac Challenges in Immune Checkpoint Therapy: Complete Heart Block With Pembrolizumab

**DOI:** 10.7759/cureus.57244

**Published:** 2024-03-30

**Authors:** Rand Sabanci, Moiz Saeed, Kevin Watat, Andrew G Kim, Dina Shaban, Georgette Nader, Harith Ghnaima, Matthew Wilcox, Fatima Ali-Ahmed

**Affiliations:** 1 Internal Medicine, Michigan State University, East Lansing, USA; 2 Internal Medicine, Michigan State University College of Human Medicine, East Lansing, USA; 3 Internal Medicine, Bronx Care, New York, USA; 4 Internal Medicine, Michigan State Sparrow Hospital, Lansing, USA; 5 Cardiology, Sparrow Hospital Thoracic and Cardiovascular Institute, Lansing, USA; 6 Electrophysiology, Sparrow Hospital Thoracic and Cardiovascular Institute, Lansing, USA

**Keywords:** pacemaker, ventricular tachycardia, pembrolizumab, renal cell carcinoma (rcc), immune check-point inhibitor, complete heart block, pembrolizumab side effect

## Abstract

Immune checkpoint inhibitors (ICIs) have revolutionized cancer treatment, yet they come with a spectrum of immune-related adverse events, including cardiac complications. We present the case of a 72-year-old male with metastatic renal cell carcinoma who developed complete heart block and ventricular arrhythmias following pembrolizumab therapy. Despite no evidence of myocarditis, the patient's condition rapidly deteriorated, ultimately resulting in his demise. This case underscores the critical need for vigilance in recognizing and managing potential cardiotoxicity associated with ICIs. Additionally, it highlights the importance of multidisciplinary collaboration in optimizing diagnostic and therapeutic strategies for patients undergoing immune checkpoint inhibitor therapy.

## Introduction

The advent of immunotherapy, particularly immune checkpoint inhibitors, has transformed the landscape of solid tumor treatment. Innovative agents targeting molecules such as the programmed death (PD) receptor or programmed death ligand 1 (PD-L1) have demonstrated significant improvements in overall survival for patients with advanced metastatic renal cancer.

Among the various immune checkpoint inhibitors (ICIs) approved for clinical use, including pembrolizumab, nivolumab, and atezolizumab, these antibodies have exhibited efficacy across a spectrum of cancers. However, their utilization has been accompanied by a diverse range of immune-related adverse events including colitis, dermatitis, endocrinopathies, hepatitis, and pneumonitis.

Our case presents a 72-year-old male patient who experienced a complete heart block followed by ventricular arrhythmias subsequent to the initiation of pembrolizumab therapy for metastatic renal cell carcinoma. This underscores the importance of vigilance and prompt recognition of potential cardiotoxicity associated with immune checkpoint inhibitors in oncological management.

## Case presentation

A 72-year-old male with a significant medical history including hypertension and stage 4 renal cell carcinoma with lung metastasis presented to the emergency department due to worsening dyspnea over the past few days, accompanied by dysphagia. He had undergone a right nephrectomy two months earlier and was currently receiving palliative immunotherapy with oral pembrolizumab and lenvatinib, initiated four weeks prior.

**Figure 1:**
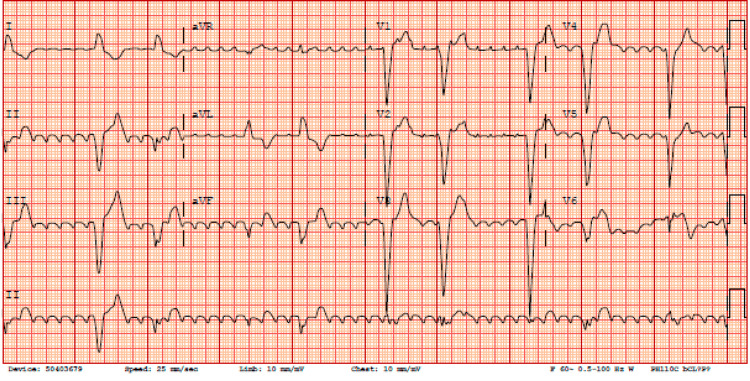
EKG showing initial atrial flutter rhythm, left bundle branch block with heart rate around 61 beats per minute.

**Figure 2:**
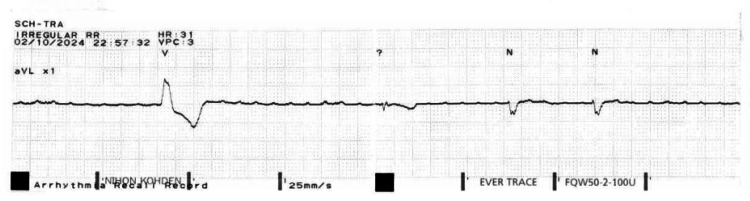
Telemetry strip showing complete heart block.

**Figure 3:**
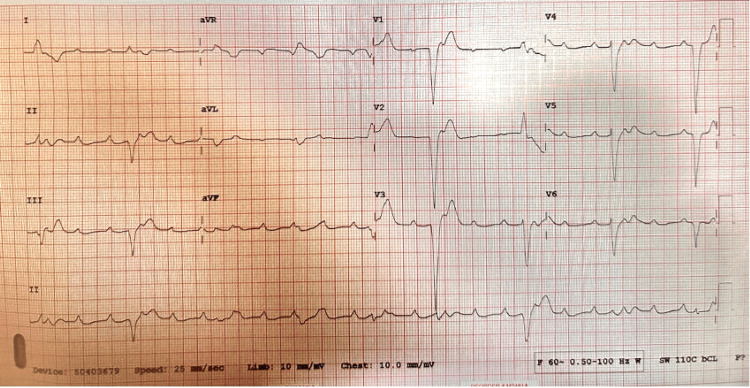
EKG showing complete heart block.

Upon examination in the emergency department, he exhibited hypoxemia necessitating noninvasive ventilation and tachycardia with a heart rate exceeding 120 beats per minute and hypotension with blood pressure around 80/55. Laboratory investigations revealed markedly elevated troponin levels of 3499 ng/L, creatinine 2.01 mg/dL, estimated glomerular filtration rate of 34 mL/min, and creatine phosphokinase (CPK) 4357 U/L. An electrocardiogram (EKG) indicated atrial flutter (Figure 1), necessitating synchronized cardioversion at 200 joules, followed by the development of a complete heart block (Figures 2, 3) unresponsive to atropine. External pacing was initiated alongside a dopamine infusion, with subsequent emergent temporary pacemaker placement.

**Figure 4:**
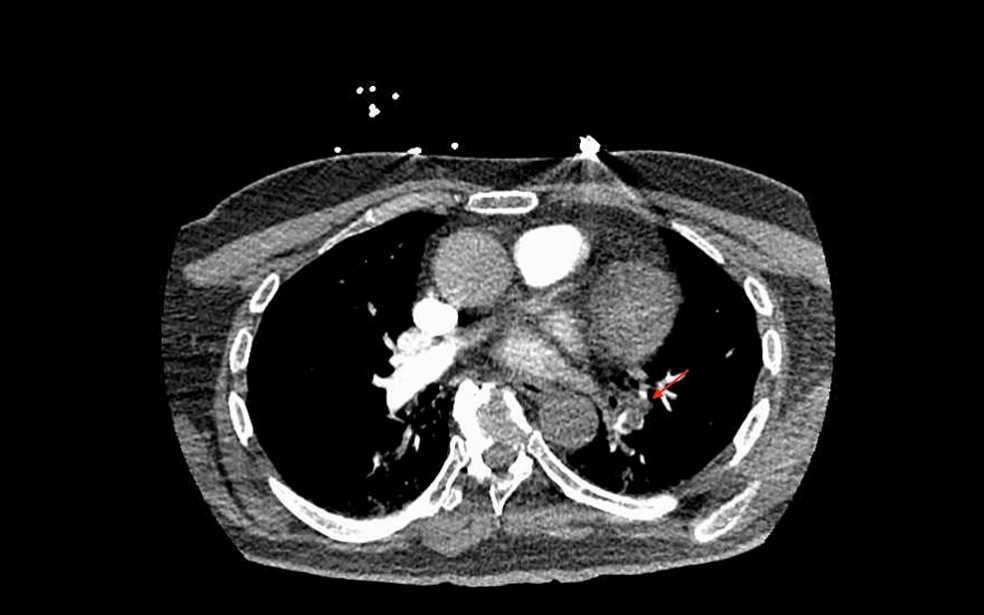
Computed tomography pulmonary angiography (CTA PE) showing left-sided segmental pulmonary embolus indicated with red arrow.

**Figure 5:**
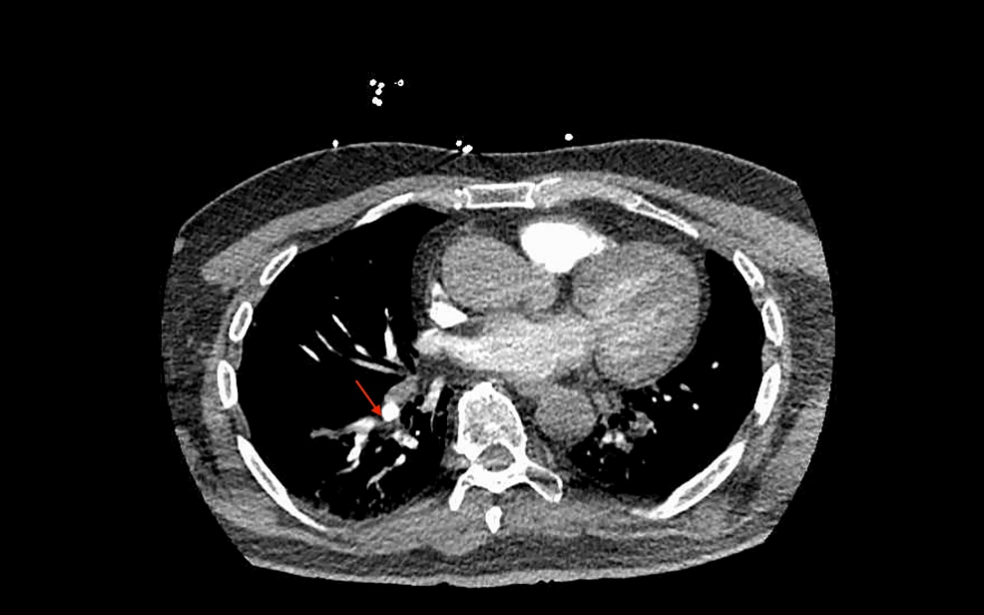
Computed tomography pulmonary angiography (CTA PE) showing right-sided segmental pulmonary embolus indicated with red arrow.

A computed tomography pulmonary angiography (CTA PE) scan revealed bilateral segmental pulmonary emboli (Figures 4, 5) prompting the initiation of a heparin infusion. Subsequently, due to declining mental status, the patient was intubated for airway protection, with escalating vasopressor support required, including dopamine, vasopressin, and norepinephrine.

Echocardiography revealed a hyperdynamic left ventricle with a preserved ejection fraction (>70%) and abnormal septal wall motion attributed to interventricular conduction delay (Video 1). The patient developed slow ventricular tachycardia with a heart rate of 130-140 beats per minute and a systolic blood pressure of 90 mmHg. Overdrive pacing was attempted to suppress the ventricular tachycardia, resulting in transient ventricular fibrillation, which spontaneously resolved within seconds. Subsequently, the patient was initiated on amiodarone.

**Video 1:** Apical four-chamber view of echocardiogram showing hyperdynamic left ventricle with preserved ejection fraction (>70%) and abnormal septal wall motion attributed to interventricular conduction delay.

Further investigation via left heart catheterization demonstrated normal coronary arteries, and plans were made for implantable cardioverter-defibrillator (ICD) placement. Following extubation, the patient experienced a pulseless electrical activity arrest. In accordance with the wishes of the patient and family, further aggressive interventions, including chest compressions or re-intubation, were withheld, and the patient passed away.

## Discussion

In recent years, ICIs have revolutionized the treatment paradigm across various cancer types. The range of malignancies amenable to ICI therapy continues to expand and includes bladder cancer, melanoma, lung cancer, renal cell carcinoma, head and neck cancers, hepatocellular carcinoma, among others [1].

Pembrolizumab, a humanized monoclonal IgG4 antibody, gained initial approval from the Food and Drug Administration (FDA) in 2014. Its indications in oncology have steadily broadened since then. Notably, the KEYNOTE-426 trial demonstrated the superior clinical outcomes of pembrolizumab plus axitinib over sunitinib, reinforcing its status as the first-line treatment for advanced renal cell carcinoma [2].

The mechanism of action of pembrolizumab involves targeting PD1, a transmembrane receptor expressed on activated lymphocytes. PD1 binds to programmed cell death protein 1 and 2 (PD-L1 and PD-L2) on other transmembrane proteins. This interaction inhibits T-cell function, and PD-L1 expression on neoplastic cells is recognized as a major mechanism of immune evasion [1].

Despite the therapeutic benefits of ICIs, they are associated with a spectrum of immune-related adverse events, including colitis, dermatitis, endocrinopathies, hepatitis, and pneumonitis [1]. Of particular concern are cardiac side effects, which have been underreported due to the lack of routine cardiac health monitoring [1].

Research, such as that conducted by Salem et al., has revealed various cardiac complications associated with ICIs, including myocarditis, pericardial disease, supraventricular arrhythmias, and heart failure [3].

Furthermore, studies like the one conducted by Brumberger et al. have shown significant differences in the occurrence of adverse cardiac events between pembrolizumab and other ICIs such as nivolumab [1]. In their research, Brumberger et al. reported a significantly higher percentage of women experiencing cardiac events compared to men (8.1% versus 2.9%), as well as a higher percentage of African Americans experiencing cardiac events compared to Caucasians (12% versus 4%) [1]. In addition, Brumberger et al. noted significant disparities in cardiac event rates between patients treated with pembrolizumab and those treated with nivolumab [1]. Specifically, patients undergoing pembrolizumab therapy exhibited a higher incidence of cardiac events compared to those receiving nivolumab (7% versus 4%). Their findings highlighted pericarditis as the most commonly reported event (2.2%), with atrial fibrillation with rapid ventricular response and heart failure occurring less frequently (2.0% and 1.5%, respectively) [1]. 

Analyzing the differences in event occurrence across malignancy types, Brumberger et al. observed that the majority of adverse cardiac events occurred in patients with non-small cell lung cancer [1]. Notably, heart failure was found to be more prevalent in patients treated with pembrolizumab, with a rate of 2.4%, compared to only 0.5% in the nivolumab group [1]. 

Our case report presents a unique instance of a patient developing conduction disorders and ventricular arrhythmias without myocarditis following pembrolizumab therapy for metastatic renal cell carcinoma. This occurrence is rare, with only a few similar cases reported in the literature. Notably, conduction disorders typically manifest within six weeks of ICI initiation similar to our case in which our patient was started on pembrolizumab four weeks prior to his admission [4]. In the case of pembrolizumab-induced complete heart block by Jang and Stream, similar to our patient, the patient was treated for metastatic renal cell carcinoma and presented with worsening exertional dyspnea [5].

The development of ventricular tachycardia in our patient underscores the potential role of an exaggerated immune response targeting the heart's electrical conduction system. Although steroid administration has shown promise in preventing ventricular tachycardia recurrence in ICI-treated patients, our patient did not receive steroids due to the lack of evidence supporting their use in ventricular arrhythmias without myocarditis [6].

Vigilance and early detection of ICI-related cardiac side effects are crucial. A comprehensive surveillance approach, including thorough cardiac risk assessment and diverse testing methods, is essential. 

## Conclusions

This case underscores the importance of establishing appropriate monitoring protocols for the increasing number of patients undergoing immune checkpoint inhibitor therapy. A multidisciplinary team comprising primary care providers, oncologists, cardiologists, and specialized cardio-pathologists is essential to optimize diagnostic and therapeutic strategies and mitigate the risk of severe immune checkpoint inhibitor-related cardiotoxicity.
